# Skin Acceptability of a Topical Cream Containing Zinc Oxide, Zinc Gluconate, Taurine and Panthenol in Females With Urinary Incontinence

**DOI:** 10.1111/jocd.71053

**Published:** 2026-08-05

**Authors:** Leonardo Celleno, Linda Landini, Dalia Mostafa Kamal, Fabrizio Mazzicone

**Affiliations:** ^1^ Eurofins Cosmetics & Personal Care Italy Srl Rome Italy; ^2^ Menarini Group Florence Italy

**Keywords:** diaper dermatitis, emollients, humectants, incontinence‐associated dermatitis, skin barrier

## Abstract

**Background:**

Incontinence‐associated dermatitis (IAD) can be exacerbated by friction and occlusion from a diaper/incontinence pad. Skin barrier function in IAD can be preserved through humectant and emollient‐containing protective agents.

**Aims:**

To investigate the efficacy, skin acceptability, and cosmetic qualities of a cream containing zinc oxide, zinc gluconate, taurine, and panthenol.

**Patients and Methods:**

Participants without IAD who used diapers included otherwise healthy females ≤ 65 years old with urinary incontinence due to surgery/a traumatic event (*n* = 11) or > 65 years old with urinary incontinence from any cause (*n* = 11). The cream was used for 28 days, applied to clean, dry skin within the diaper area at each diaper change/as needed. Participants noted daily any reactions and sensations of discomfort. The investigator conducted a visual clinical examination of the skin around the application area at study start (D0) and after 28 days (D28), noting at D28 any reactivity (location, intensity, percentage) and product intolerance. Participants reported any discomfort and rated product efficacy and tolerability.

**Results:**

At D28, no participant had clinical signs or sensations of discomfort or experienced a treatment‐related adverse event. The product was rated by the dermatologist as ‘very good’ in terms of skin acceptability and tolerance. Most participants agreed the product had good moisturizing efficacy, helped alleviate redness and irritation, and made their skin feel protected and comfortable.

**Conclusion:**

The investigational cream containing zinc oxide, zinc gluconate, panthenol, and taurine was generally well‐tolerated. Further investigations, including a control group, are needed to determine if the cream prevents IAD.

## 1 Introduction

Urinary incontinence occurs in approximately 30% of women and 20% of men, with experience of such varying widely due to incontinence type, age, weight, and risk factors [1, 2]. In middle‐aged women, stress incontinence, the most common form of urinary incontinence, can particularly occur postpartum or due to trauma or surgery. It occurs due to high urethral mobility; internal urethral sphincter dysfunction; or urethral ligament, fascia, and pelvic floor muscle relaxation [3]. In men, urinary incontinence may be due to conditions such as benign prostate enlargement or inflammation; prostate cancer; detrusor muscle weakness or overactivity; or a urinary tract infection [4]. Urinary incontinence can be detrimental to quality of life and be the cause of stigmatization and situational worry, depression, and anxiety [3, 4, 5, 6]; however, it is frequently underreported to a general practitioner (GP), especially in younger age groups [7].

Incontinence‐associated dermatitis (IAD), which can be a form of both irritant and contact dermatitis, can occur due to chronic exposure to urine or stool, a more alkaline pH environment, and overhydration. Combined with friction and occlusion from a diaper/incontinence pad, these can lead to loss of skin barrier function, infection, and stratum corneum damage [8, 9, 10, 11, 12, 13]. IAD is characterized by erythematous, inflamed skin, which may be accompanied by superficial layer erosion, partial‐thickness wounds, and secondary cutaneous infection. It can occur both in the immediate genital and anal areas as well as across the buttocks and thighs and in skin folds and beyond in contact with diapers/incontinence pads, especially in cases where there is also candidiasis [10, 13, 14, 15, 16].

Diagnosis of IAD is typically through visual inspection, revealing shiny skin that is much redder than usual and/or is hypopigmented in darker skins. Compared to pressure ulcers, IAD is more typically limited to genital and diaper/incontinence pad areas, occurs only at the dermal/epidermal level, and does not lead to tissue necrosis [16]. Studies of IAD prevalence in people who are incontinent vary widely, with an occurrence of around 5% typical for people in longer term settings, and with occurrence of around 40% in acute care [17, 18, 19, 20].

For older adults, IAD may be exacerbated by myriad age‐associated skin changes [13, 21] such as loss of elastin, dermal tissue bulk, and overall cell number; reduction in epidermal turnover, skin hydration, and sebum production; increasing space between collagen bundles; and papillae flattening and weakening of papillae to epidermis connections [20, 22, 23]. The result is skin that is more fragile and more tear‐ and injury‐prone. Combined with these factors is a decrease in immune system function that can contribute to skin barrier function effectiveness. These can also lead to an increase in sensitivity to irritants, including urine and stool [20, 22, 23].

Treatment of IAD focuses on three main goals: (i) removal of irritants from the affected skin; (ii) eradication of cutaneous infections such as candidiasis; and (iii) urine and stool containment or diversion. Along with the use of appropriately absorbent, breathable diapers/incontinence pads and gentle cleansing when needed, skin barrier function can be preserved through the use of protective agents and emollients [14, 15, 16, 23, 24, 25]. Such products should contain humectants to attract water to the stratum corneum and emollients to replace stratum corneum intercellular lipids and help preserve skin barrier integrity [15]. While antimicrobial and corticosteroid treatments can quickly treat IAD, there use is not advised long‐term due to the adverse event (AE) profiles of these agents [26].

This current study examined the use of a cream containing zinc oxide, zinc gluconate, taurine, and panthenol. Zinc oxide, a recommended skin‐protecting ingredient [15, 18, 23], has very low water solubility and is used in a number of dermatological conditions including contact and irritant dermatitis. It has antibacterial properties [27] and can reduce levels of IAD and inflammatory markers [28]. Zinc gluconate, the water‐soluble zinc salt of gluconic acid, has been used in anti‐acne treatments [29] and to aid wound healing [30], but its exact mechanism of action is yet to be elucidated.

Taurine, an essential amino acid, is involved in stimulation of skin barrier lipid synthesis [31] and maintenance of keratinocyte hydration [31], which can lead to preservation of epidermal barrier function following insult [31]. Taurine use can reduce indices of inflammation and encourage new epithelial formation [32]. Panthenol, a hygroscopic moisturizer and humectant widely used in skin care and wound treatment, is easily absorbed through the skin, where it is converted to pantothenic acid [33], a molecule essential to epithelial function [34]. Use of panthenol can reduce transepidermal water loss and improve stratum corneum hydration [35].

This study intended to confirm the efficacy, skin acceptability, and cosmetic qualities of a zinc oxide, zinc gluconate, taurine, and panthenol cream for prevention of diaper dermatitis over 28 consecutive days of repeated application. This was carried out in a group of female participants who had urinary incontinence and used diapers. Half of the group were ≤ 65 years old and had such incontinence due to surgery or a traumatic event. Participants did not have IAD at study entry and there was no control treatment.

## 2 Material and Methods

### 2.1 Participants

This open‐label, monocentric clinical study was performed at the Institute of Eurofins Cosmetics & Personal Care Italy, Srl, in accordance with the principles of the Declaration of Helsinki and Good Clinical Practices for conducting clinical trials of drugs. The protocol, informed consent form, and preclinical information concerning the investigational product (particularly regarding its safety) were approved by the investigating center's Internal Revision Committee. Study data are available upon request.

Participants were drawn from a general panel held by the investigating center. All participants gave written, informed consent prior to study start. Inclusion criteria included otherwise healthy females (as declared at birth) with Fitzpatrick phototypes I–IV. There were two groups of participants: one was aged ≤ 65 years and had urinary incontinence derived from surgery or a traumatic event (e.g., gynecological disorders, postpartum), the other was aged > 65 years and had urinary incontinence from any cause. Participants did not exhibit any signs or symptoms of IAD at study start.

Exclusion criteria included having received a vaccination within 2 weeks prior to study start or intending to be vaccinated during the course of the study; planned hospitalization during the study; a history of an adverse reaction to the same type of products as the investigational product; use of systemic retinoids or desensitization treatment within 6 months of study start; use of antibiotics or topical or systemic medication with anti‐inflammatory or antihistamine products within 2 weeks of study start; therapy for cancer within 5 years of study start; breastfeeding, pregnant, or planning a pregnancy during the study; started or changed estrogen‐progesterone contraception or hormonal treatment (including such treatment prescribed to reduce acne with cyproterone, drospirenone, or norgestimate) within the 3 months prior to the study or foreseeing use for the study duration.

During the study, participants could not use drugs that could interfere with the study and the interpretation of the results (e.g., systemic or topical anti‐acne medication; anti‐acne cosmetic products; topical or systemic medication with anti‐inflammatory or antihistamine properties; antibiotics; desensitization treatment); have an aesthetic procedure on the experimental area (e.g., laser, depilation); have a significant change in lifestyle (e.g., diet, smoking, sport); use any cosmetic product similar to the tested one; or change their usual hygiene products or introduce new cosmetic products.

### 2.2 Test Product

The test product was Relizema baby care (RELIFE MENARINI group, Florence, Italy) with ingredients including 6% zinc oxide, zinc gluconate, panthenol, and taurine, in a ‘4‐Pro system’. Other ingredients include *prunus amygdalus dulcis* (almond oil), dimethicone, glycerin, and *butyrospermum parkii* (shea) butter, as well as other antimicrobial, lubricating, emollient, fragrance, carrying, and wetting agents. The product was supplied in neutral packaging. The cream was applied at home for 28 consecutive days. Participants were instructed to apply a layer of cream to clean and dry skin within the diaper area as part of each diaper change or as needed.

### 2.3 Study Procedure

A visual clinical examination of the skin around the application area was performed at the investigating center by the same dermatologist for all participants, under a standard ‘daylight’ source, at the start of the study (D0) and after 28 consecutive days of product use (D28). Participants noted in a daily log any reaction observed and any sensations of discomfort. They could use their own words to express what they felt.

In cases of any reactivity noted at D28, the dermatologist noted the clinical signs (e.g., erythema, oedema, dryness/desquamation) and any sensations of discomfort declared by the participants (e.g., heating, burning, stinging, itching, pulling, redness); the location, duration, period of occurrence after product application; the frequency, intensity, and evolution of the condition; and any medical treatment undertaken. The dermatologist also calculated the percentage of the reaction. Intensity was assessed according to ordinal scales devised for the study (Table 1). The investigational product as the cause of an incidence of reactivity was assessed as being ‘excluded,’ ‘unlikely,’ ‘not clearly attributable,’ ‘likely,’ or ‘very likely’. Digital photographs of the skin were systematically taken in the case of an AE. AEs included any harmful or unwanted reaction due to the investigational product, including any slight rection of intolerance or sensation of discomfort.

**TABLE 1:** Ordinal scales to assess the intensity of the main clinical signs and sensations of skin discomfort.

Condition	Score	Condition	Score
Erythema	0: None	Papule	0: None
	1: Very slight		1: Few
	2: Slight		2: Numerous
	3: Moderate	Pustule	0: None
	4: Severe		1: Few
Skin oedema	0: None		2: Numerous
	1: Slight	Nodule	0: None
	2: Moderate		1: Few
	3: Severe		2: Numerous
Skin dryness	0: None	Comedo	0: None
	1: Slight		1: Few
	2: Moderate		2: Numerous
	3: Important	Vesicle	0: None
Sensations of discomfort	0: None		1: Few
	1: Very slight		2: Numerous
	2: Slight		
	3: Moderate		
	4: Severe		

All the data were recorded in the case report form and the participant was anonymized.

At the end of the test period, the same dermatologist rated the skin acceptability (tolerance) of the investigational product based, separately, on ‘percentage of test participants exhibiting clinical signs attributable to the investigational product’ and ‘percentage of test participants exhibiting sensations of discomfort attributable to the investigational product.’ Ratings with their respective percentages were: ‘very good’ (0%, 0%), ‘good’ (0%, < 30%), ‘moderate’ (< 20%, any percentage; or 0%, 30%–50%), or ‘bad’ (≥ 20%, any percentage; or 0%, > 50%).

The observance of the experimental conditions by the test participants was assessed using a questionnaire at D28, devised by the study center. Participants rated from 0 to 3 (‘disagree,’ ‘slightly disagree,’ ‘slightly agree,’ ‘agree’) if the investigational product: ‘had a good moisturizing efficacy’; ‘made the skin soft and velvety’; ‘had a good soothing effect and provided relief’; ‘helped alleviate redness and irritation caused by diapers’; made the participant ‘feel refreshed and/or the product refreshed the participant's skin’; made the participant's skin ‘feel protected and more comfortable’; ‘didn't make the participant feel sticky’. Participants were also asked about the way they used the investigational product, so that the investigator could assess possible deviations from the experimental conditions.

The scales used for the evaluations, while similar to those commonly used in this type of study, do not originate from officially validated assessment tools. The study was only intended to assess the skin acceptability of the investigational product and to appreciate its cosmetic qualities and efficacy in a panel of otherwise healthy human participants with urinary incontinence after repeated applications, under normal conditions of use, for 28 consecutive days. For this reason, no statistical analysis of the results was conducted; instead, only the percentage of positive or negative responses was reported and formal statistical comparison was not carried out between the groups.

## 3 Results

The study was conducted between March 10th, 2023 and April 7th, 2023. Twenty‐two female participants were recruited and all completed the study. As can be seen from Table 2, half of the participants were ≤ 65 years old [mean age (range): 59.5 (54–65) years], all of whom had urinary incontinence derived from surgery or traumatic events. The other participants were > 65 years old [mean age (range): 72.1 (66–88) years] and had urinary incontinence from any cause. All participants had skin phototypes II or III and all lived at home. Seven participants received help from a caregiver to apply the study cream.

**TABLE 2:** Participant demographics.

Study number	Age	Fitzpatrick phototype	UI from surgery/traumatic event	Current medication	Medical condition
1	56	III	Yes	None	None
2	63	III	Yes	Pantoprazole	GERD
3	58	III	Yes	Levothyroxine/metformin	Hypothyroidism/diabetes
4	54	II	Yes	None	None
5	70	II	No	Metformin	Diabetes
6	65	II	Yes	None	None
7	57	III	Yes	None	None
8	65	III	Yes	Semaglutide/insulin	Diabetes
9	63	III	Yes	None	None
10	88	II	No	Olmesartan	Hypertension
11	87	III	No	Pantoprazole/Multivitamin	GERD/osteoporosis
12	59	III	Yes	None	None
13	61	III	Yes	Levothyroxine/omeprazole	Hypothyroidism/GERD
14	88	II	No	Lithium/omeprazole	Anxiety/GERD
15	66	III	No	None	None
16	85	II	No	Levothyroxine	Hypothyroidism
17	86	II	No	Perindopril arginine/amlodipine	Hypertension
18	54	III	Yes	None	None
19	86	II	No	Ramipril	Hypertension
20	76	III	No	Metformin/pantoprazole	Diabetes/GERD
21	80	II	No	Beclomethasone/ formoterol/glycopyrronium bromide	Asthma
22	78	III	No	Metformin	Diabetes

Abbreviations: GERD, gastroesophageal reflux disease; UI, urinary incontinence.

As assessed by the dermatologist, all participants had clear skin on D0 and none reported any sensations of discomfort. At D28, none of the participants had clinical signs or reported sensations of discomfort attributable to the investigational product. No AE related to the investigational product was reported by the participant or observed by the dermatologist, who rated the investigational product as having ‘very good’ skin acceptability and tolerance.

As shown in Table 3, for all the participant‐rated questions regarding the products' moisturizing, soothing, and protecting factors, 81.8% of test participants gave ratings of ‘agree’ and 13.6% of ‘slightly agree.’ This was considered significant according to the analysis plan. Two of the three participants who ‘slightly agreed’ with each statement were from the surgery/trauma‐derived incontinence group, the other was from the > 65 year old group (aged 86). The one participant who ‘slightly disagreed’ with all statements was in the > 65 year old group (aged 80).

**TABLE 3:** Participant‐rated responses (*n* = 22).

Question: The product	Slightly disagree	Slightly agree	Agree
has a good moisturizing efficacy	1 (4.6%)	3 (13.6%)	18 (81.8%)
makes the skin soft and velvety	1 (4.6%)	3 (13.6%)	18 (81.8%)
has a good soothing effect and gives me relief	1 (4.6%)	3 (13.6%)	18 (81.8%)
helps to alleviate redness and irritation caused by diapers	1 (4.6%)	3 (13.6%)	18 (81.8%)
makes me feel refreshed and/or the product refreshes your skin	1 (4.6%)	3 (13.6%)	18 (81.8%)
makes my skin feel protected and more comfortable	1 (4.6%)	3 (13.6%)	18 (81.8%)
doesn't make me feel sticky	1 (4.6%)	3 (13.6%)	18 (81.8%)

## 4 Discussion

As important as treating IAD when it occurs is trying to prevent IAD occurrence [11, 13, 36, 37]. This is because, while at first symptoms may be just uncomfortable, skin damage associated with IAD can lead to exposure to pathogens; excessive, painful ulceration; secondary infections; and subsequent considerable healthcare costs and quality of life impacts [11, 24].

In this preliminary, monocentric study, while it was not designed to assess response of IAD, the experimental conditions adopted, such as the application area, quantity of investigational product applied and frequency of application, reproduced the normal conditions of use advocated for creams to protect skin from IAD [11, 13, 36, 37]. The cream used here is an over‐the‐counter formulation. Such access may be of use for people with IAD as urinary incontinence is a condition people may not discuss with their GP [7].

In this study, participants' skin was clear of IAD at baseline, as assessed by a trained evaluator. Following 28 days' use of the cream, dermatological assessment found no development of an AE associated with the formulation or of evidence of IAD. Participants indicated that the product was acceptable with regard to factors such as moisturizing efficacy and skin softening, soothing, and protection. It was also agreed that the product did not make them feel sticky and that it helped to alleviate redness and irritation caused by diapers. It is of note that these participant‐recorded effects were only measured subjectively, which may have limitations due to personal interpretations of what each factor means and by how much it changed, where relevant. Further studies could ascertain levels of previous redness and irritation and prior experience of IAD and could integrate objective means to assess product properties such as effects of the formulation on transepidermal water loss.

The formulation used in this study has several ingredients, including panthenol, zinc oxide, taurine, and zinc gluconate. These ingredients were chosen due to prior studies showing their efficacy. For example, in people with irritant or allergic contact dermatitis, the use of creams containing dexpanthenol (5%)—which, when converted to pantothenic acid, is involved in epithelial function [33, 34]—can lead to significant decreases in eczematous or xerotic symptoms with good skin tolerability after up to 28 days' use [35]. AEs following topical use of dexpanthenol, such as an allergic reaction, are reported as rare [35, 38, 39].

Zinc oxide may also be of use for alleviating IAD with one study where a zinc oxide‐containing cream was used in ward‐based patients with urinary and/or fecal incontinence showing IAD healing in around a third of patients, with little skin deterioration [40]. Dexpanthenol and zinc oxide in combination were used in a 7‐day investigation involving children with stool‐induced irritant diaper dermatitis, compared with the ointment base. Here, both treatments led to significant decreases from baseline in transepidermal water loss, with a significant advantage for the formulation with the active ingredients at Day 3, along with decreases in severity score, with no difference between the two formulations at Day 7 [41].

While the amino acid taurine has a known role in skin cell and layer maintenance and repair [31], clinical investigation into its use for dermatological conditions is still rare. In vitro investigations have found that topically applied taurine can accumulate in keratinocytes and provide protection against osmotically induced apoptosis [42], and, following an induced insult, taurine can stimulate barrier lipids in an epidermis model [31]. One clinical study showed that adding 0.1% taurine and 0.1% aloe to an aluminum chloride (AlCl_3_)‐based antiperspirant reduced measures of irritant contact dermatitis attributed to AlCl_3_ [43]. Lastly, a recent study of the actions of zinc gluconate in a mouse model of atopic dermatitis found that injection of this ingredient led to significant lowering of associated skin symptoms [44].

The combination of these four active ingredients has also been tested. In infants with IAD, use of this formulation was shown to reduce erythema [45] and provide protective efficacy, and was acceptable to users [46]. In three other studies of this formulation, that together included 20 infants aged 3–36 months with skin erythema, 28 days' use led to clearance of this condition in 80% of participants at Day 14 and all participants by study end. The infants' caregivers were all satisfied with the cream's moisturizing efficacy, softening effects on skin, and ease of application [47, 48, 49].

Another study, which included 10 adults (mean age 84 ± 6.2 years) and 10 infants (aged 1 day to 4 years) with mild‐to‐moderate IAD, showed that 30 days' use of the combination cream led to a significant decrease in clinical erythema assessment and improvement in investigator global assessment scores in both groups. Product tolerability was rated as excellent in 90% of patients [50]. A further study, with a barrier spray containing similar ingredients to the one used currently, found it displayed utility in treating symptoms of mild–moderate intertrigo with regards to erythema and pruritus. The product was generally well‐tolerated with no AEs reported [51].

While this study helps confirm the utility of the test product, study limitations include its monocentric design. Further investigation could include a control condition, such as use of the cream versus no cream, a non‐active ingredient cream, or a different IAD‐alleviating treatment. Other study limitations are that the participant numbers were low, that they were all female, and that they all had skin with Fitzpatrick phototype II–III. As such, current results, while valid, may predominantly apply to similar patients. Further studies could include a larger number of patients, more than a single gender, and a fuller range of skin phototypes to assess product use across a wider spectrum of users.

## 5 Conclusion

Overall, this study showed that a cream containing zinc oxide, zinc gluconate, panthenol, and taurine was generally well‐tolerated. Further investigations could include a control condition, people with IAD, and a wider demographic array of participants.

## Author Contributions

L.C. was an employee of Eurofins Cosmetic and Personal Care Italy. D.M.K. and L.L. were involved in study design and administration. F.M. was involved in manuscript coordination and administration following the passing of Prof. L.C.

## Funding

This study was funded by Menarini Group.

## Disclosure

The protocol, informed consent form, and preclinical information concerning the investigational product (particularly regarding its safety) were approved by the investigating center's Internal Revision Committee on February 28th, 2023.

The study was performed in the spirit of:

- The general principles of medical ethics in clinical research coming from the Declaration of Helsinki (June 1964) and its successive amendments.
- The international recommendations relating to Good Clinical Practices for conducting clinical trials for drugs ICH TOPIC E6 (R2) of November 2016 (CPMP/ICH/135/95)
- The Directive of the European Parliament and Council 2001/20/EC concerning the harmonization of legislative, statutory and administrative provisions of the member States relating to the application of good clinical practices when conducting clinical trials for drugs for human use—OJ/EC of 01/05/2001
- The Regulation (EC) No 1223/2009 of the European Parliament and of the Council of 30 November 2009 on cosmetic products.
- The Commission Regulation (EU) No 655/2013 of 10 July 2013 laying down common criteria for the justification of claims used in relation to cosmetic products.
- The recommendations of Colipa—August 1997: “guidelines for the assessment of human skin compatibility”
- The Regulation (EU) 2016/679 of the European Parliament and of the Council of 27 April 2016 on the protection of natural persons with regard to the processing of personal data and on the free movement of such data, and repealing Directive 95/46/EC (General Data Protection Regulation).

## Conflicts of Interest

L.C. was an employee of Eurofins Cosmetic & Personal Care Italy. D.M.K., L.L., and F.M. were all employees of the Menarini Group at the time of initial manuscript’s submission.

## Data Availability

The data that support the findings of this study are available from the corresponding author upon reasonable request.
